# Huperzine A suppresses absence seizures in the genetic absence epilepsy rat from Strasbourg (GAERS) model of genetic generalized epilepsy with absence seizures

**DOI:** 10.1002/epi4.13016

**Published:** 2024-08-03

**Authors:** Pablo M. Casillas‐Espinosa, Jennie Garcia‐Olivares, Rui Li, Crystal Li, Chungping Yu, Andrea E. Formella, Terence J. O'Brien

**Affiliations:** ^1^ Department of Medicine The Royal Melbourne Hospital, The University of Melbourne Parkville Victoria Australia; ^2^ Department of Neuroscience, School of Translational Medicine, Monash University Melbourne Victoria Australia; ^3^ Department of Neurology The Alfred Hospital Melbourne Victoria Australia; ^4^ Supernus Pharmaceuticals, Inc. Rockville Maryland USA

**Keywords:** crossover study, ethosuximide, GAERS model, idiopathic generalized epilepsy, spike–wave discharges, SPN‐817

## Abstract

**Objective:**

We evaluated huperzine A treatment in the Genetic Absence Epilepsy Rat from Strasbourg (GAERS) model of genetic generalized epilepsy (GGE) with absence seizures.

**Methods:**

Adult male GAERS (*N* = 15) were implanted with EEG recording electrodes 10 days before receiving study drug. Each animal received the following six treatments as a single, intraperitoneal dose, 7 days apart (in random order): huperzine A (0.3, 1.0, or 3.0 mg/kg), two periods of vehicle (0.9% NaCl), or ethosuximide (100 mg/kg) as a positive control. Electroencephalograms (EEGs) were acquired for 24 h before and after each treatment and analyzed for seizure activity during the 90‐min period immediately post‐treatment, including 30‐min intervals at 30, 60, and 90 min. Additional analyses evaluated seizure activity over the 24‐h post‐treatment period using 60‐min intervals at 6, 12, and 24 h. The cumulative 24‐h periods before and after each administered treatment were also compared.

**Results:**

Two‐way ANOVA showed a treatment difference [*F*_(91,182)_ = 3.592, *p* < 0.0001] on the number of seizures over the first 90‐min post‐treatment (primary outcome); Tukey's post hoc analyses showed that, compared to vehicle, huperzine A (3.0 mg/kg) significantly reduced seizures in the 30‐min (*p* = 0.02) and 60‐min (*p* = 0.001) intervals, and ethosuximide significantly reduced seizures at all measured time intervals except the 1‐h blocks at 12 and 24 h. Huperzine A 3.0 mg/kg and ethosuximide significantly reduced seizures during the cumulative 24‐h post‐treatment period relative to pretreatment baseline. While huperzine A 3.0 mg/kg did not differ significantly from ethosuximide at any time point, the study was not designed to evaluate non‐inferiority. The only adverse event after huperzine A or ethosuximide was mild, dose‐dependent sedation.

**Significance:**

Huperzine A potently suppressed absence‐like seizures in GAERS, albeit with a shorter duration of action relative to ethosuximide, showing promise for clinical efficacy in GGE.

**Plain Language Summary:**

This study looked at how huperzine A affects seizures in rats with similar abnormal brain activity as seen in humans with absence epilepsy. Rats received different treatments, placebo (i.e., saline solution), huperzine A, and ethosuximide. Ethosuximide is considered a gold standard treatment for absence epilepsy. We recorded brain activity to measure seizures before and after each treatment. We found that huperzine A (3.0 mg/kg) reduced seizures soon after treatment, like ethosuximide. Both treatments appeared safe, causing only mild sleepiness. The study shows that huperzine A could be a good new treatment for a type of absence epilepsy.

**Key points**

- A single (3.0 mg/kg) dose of huperzine A suppressed seizures in the GAERS model of GGE with absence seizures.
- Ethosuximide maintained separation from vehicle at later time points than huperzine A, perhaps owing to differences in half‐life.
- Huperzine A effects did not differ significantly from ethosuximide; however, the study was not designed to evaluate non‐inferiority to ethosuximide.
- Adverse events with tested doses of huperzine A and ethosuximide were limited to mild, dose‐dependent sedation.
- Huperzine A shows promise as an anti‐seizure treatment for GGE. Future studies should evaluate chronic administration on seizure suppression.

## 1 INTRODUCTION

Absence seizures are one of the most common seizure types in patients with genetic generalized epilepsy (GGE). Absence seizures usually start during childhood and are characterized by generalized spike–wave discharges (SWDs) on electroencephalograms (EEGs).^1^ The most efficacious anti‐seizure medications for absence seizures are ethosuximide, valproic acid, and lamotrigine.^1^ However, these drugs often are not well tolerated and/or may not reduce seizure burden optimally. Moreover, the predominantly younger age of presentation and difficulty quantifying seizure burden make human clinical trials challenging.

The Genetic Absence Epilepsy Rat from Strasbourg (GAERS)^2^ is a well‐validated model of GGE with absence seizures. The GAERS model shows spontaneous absence‐like seizures with SWDs on EEGs, and typified polygenic etiology, clinical, and pharmacological characteristics of the human absence epilepsies, thus making it an ideal tool for studying this seizure type.^2^ Anti‐seizure efficacy in GAERS has proven highly predictive of anti‐seizure efficacy in human GGE with absence seizures.^2^ For example, ethosuximide, valproate, and lamotrigine suppress seizures in GAERS, whereas carbamazepine and phenytoin exacerbate seizures in these animals as they often do with absence seizures in humans.^2^ The expected high predictive validity of the GAERS model makes it ideally suited for evaluating the potential clinical utility of novel anti‐seizure drugs with varied mechanisms of action.

The objective of the current study was to evaluate (−)‐huperzine A, a compound currently in development for the treatment of focal seizures (SPN‐817 program; Supernus Pharmaceuticals, Inc.) in the GAERS model. Huperzine A is a sesquiterpene alkaloid compound found naturally in the fir moss herb *Huperzia Serrata*.^3^ It is a potent, reversible, and specific inhibitor of acetylcholinesterase (AChE) and has long been used in Chinese medicine to treat cognitive and inflammatory disorders.^3^ AChE is located in the synaptic cleft and catabolizes the neurotransmitter acetylcholine (ACh) after synaptic release. The action of AChE is critical for the termination of the synaptic signal and regulates the strength of cholinergic neurotransmission. In the central nervous system (CNS), the cholinergic system modulates neuronal excitability, neuronal network oscillations, memory and learning processes, arousal, and anti‐inflammatory responses.^4^

Anti‐seizure effects of huperzine A have been demonstrated in a variety of preclinical models of inducible seizures (e.g., 6‐Hz, pentylenetetrazol, maximal electroshock, kainic acid, N‐methyl‐D‐aspartate, hyperthermia) in rodents, and in genetic models of developmental epileptic encephalopathy, including experiments conducted on *Scn1A* and *Scn8a* mutant mice with genetically derived seizures.^3^, ^5^, ^6^, ^7^

The potential mechanisms of huperzine A anti‐seizure effects are still under investigation. Huperzine A primarily targets AChE with high selectivity for tetrameric (synaptic) AChE. Knowledge regarding the role of cholinergic neurotransmission in the manifestation and treatment of seizures is limited. In the brain, ACh acts as a neuromodulator and may promote excitatory or inhibitory effects depending on site of action, target receptor subtype, and the baseline state of neural networks.^4^, ^8^ Huperzine A is thought to avert seizures by augmenting inhibitory tone.^6^, ^9^ Additionally, the molecule's broad effects on neuronal activity, neuroprotection, and reduction of neuroinflammation (whether or not mediated by increased synaptic ACh or other mechanisms) could contribute to its anti‐seizure efficacy.

There is no evidence demonstrating that huperzine A increases GABA release or acts directly on GABA receptors. Previous research has demonstrated that certain classes of GABAergic drugs can aggravate absence epilepsy. For instance, tiagabine (GABA reuptake inhibitor) and vigabatrin (irreversible inhibitor of GABA transaminase) nonselectively potentiate GABA and aggravate absence seizures in humans and GAERS.^10^ Given the novel cholinergic mechanism of action for huperzine A as an anti‐seizure medication, the current study provided the opportunity to inform whether huperzine A might reduce seizures in patients with GGE, or alternatively exacerbate them as typically observed with many GABAergic anti‐seizure drugs.^11^

## 2 METHODS

### 2.1 Study design and treatments

**FIGURE 1:**
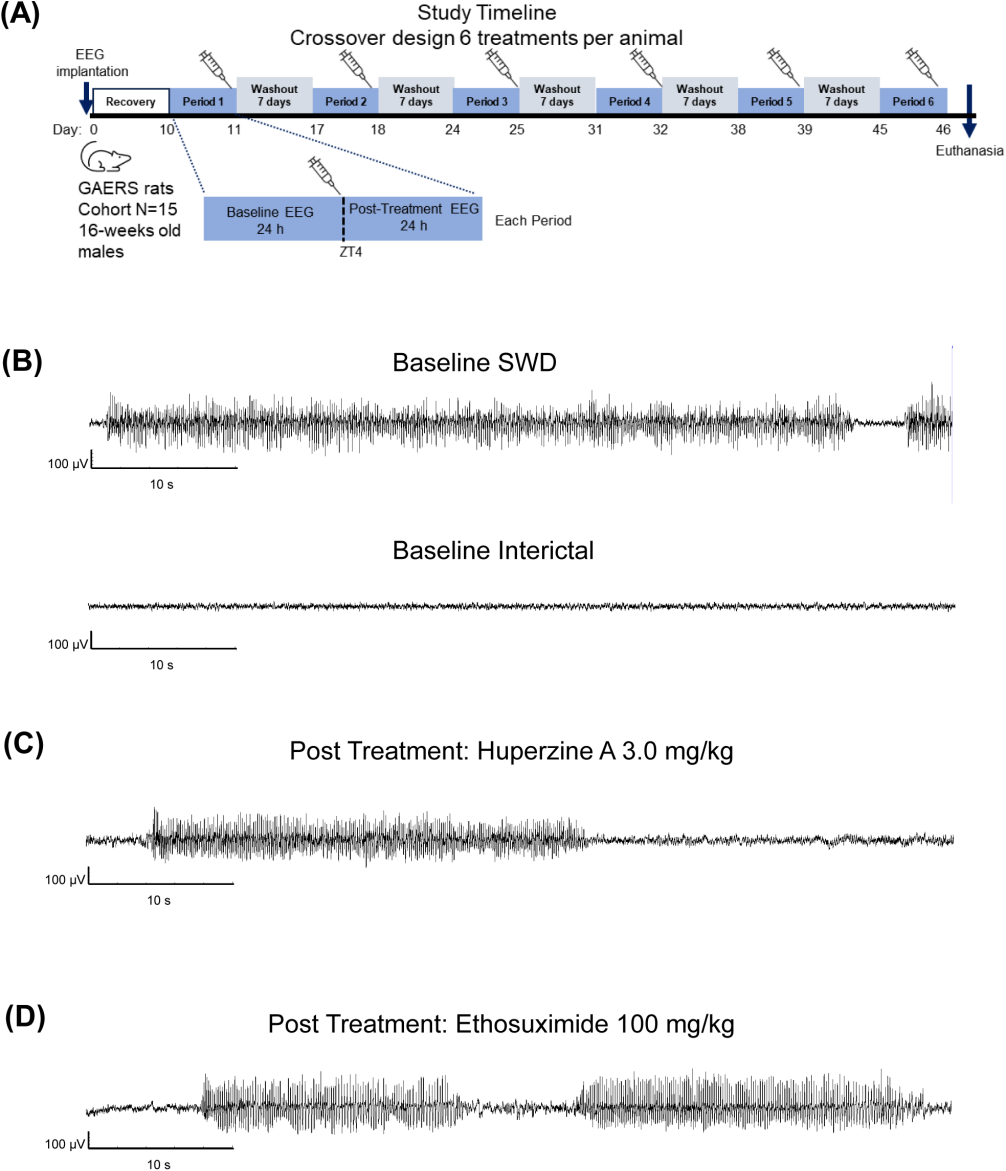
Study design and representative EEG recordings of SWDs. (A) Experimental timeline of crossover design to assess anti‐seizure activity of acute treatment with huperzine A in the GAERS model of GGE with absence seizures. (B) Representative EEG recordings of SWD and interictal periods for vehicle before treatment (baseline). (C, D) Representative EEG recordings after intraperitoneal administration of huperzine A 3.0 mg/kg (C) and ethosuximide 100 mg/kg (D). GAERS, Genetic Absence Epilepsy Rat from Strasbourg; GGE, genetic generalized epilepsy; SWD, spike–wave discharges.

A crossover design was used to minimize inter‐animal variability in seizure frequency and allow for a smaller sample size.^12^ Treatments were administered at the same time of the day (Zeitgeber time 4 [ZT4]) to avoid circadian fluctuations. Each animal received six intraperitoneal (i.p.) treatments as a bolus dose, in random order (assigned using a custom R randomizer script): three doses of huperzine A (0.3, 1.0, and 3.0 mg/kg), ethosuximide (100 mg/kg) as a positive control, and two periods of vehicle treatment to further control for variability in seizure expression across the study. Treatments were spaced 7 days apart to avoid carryover effects (the half‐life of huperzine A is ~2 h and the half‐life of ethosuximide is ~12 h). Huperzine A has shown anti‐seizure activity in different seizure models in rodents at doses of up to 1 mg/kg;^6^, ^13^ the 3.0 mg/kg dose was selected based on the maximum tolerated oral dose of 6 mg/kg in male rats.^14^ Ethosuximide was used as a positive control because it is a first‐line therapy for absence seizures in people with epilepsy^15^, ^16^, ^17^ and also has potent anti‐seizure effects in the GAERS model.^18^ The study design is illustrated in Figure 1A.

### 2.2 Animals and ethics statement

All procedures were conducted in 16‐week‐old male GAERS^3^ (*N* = 15; from The Monash Animal Research Platform, Clayton, Victoria, Australia). The study was approved by the Alfred Research Alliance Animal Ethics Committee (E/2034/2020/M) in adherence with the Australian code for the care and use of animals for scientific purposes. The animals were individually housed in alternating 12‐h cycles of light and dark. Food and water were provided ad libitum for the study duration.

### 2.3 Compound formulation

Vehicle was prepared using sterile normal saline (0.9% NaCl, pH 5.0) and was used to dissolve ethosuximide (Sigma‐Aldrich Australian Cat #E7138‐100G, Lot #MKCJ7744) at a concentration of 100 mg/mL and huperzine A (Supernus Pharmaceuticals, Inc., USA, lot #800272230) at concentrations of 0.3 mg/mL, 1.0 mg/mL, and 3.0 mg/mL.

### 2.4 EEG electrode implantation surgery

EEG electrode implantation surgery was performed at day 0 under anesthesia using methodology previously described.^3^, ^19^ Briefly, animals were anesthetized using 5% isoflurane, and Polyvisc was applied to the eyes. The skull fur was shaved, and the skin was cleaned. A midline incision was made on the scalp, and four burr holes were drilled through the skull without penetrating the dura: one on each side of the frontoparietal region (AP: ±1.7; ML: −2.5) and two on each side of the temporal region (AP: ±5. 6; ML: left 2.5). Four epidural stainless‐steel screw recording electrodes (EM12/20/SPC) were screwed into the burr holes. Ground and reference epidural stainless‐steel screw electrodes were implanted on each side of the parietal bone above the cerebellum. Recording electrodes were fixed in position by applying self‐curing dental cement, and the incision was then sutured.

Immediately after surgery, each animal received an injection of buprenorphine (0.05 mg/kg i.p.) for analgesia and 3 mL of 0.9% NaCl solution every 12 h for 2 days to prevent dehydration. The animals were allowed to remain on a heat pad overnight. Rat mush (40% coarse ground rat pellet, 40% rodent milk powder, and 20% sterile water) was given to the animals for up to 5 days. The weight, grooming, and movement of the animals were monitored every 8 h for the first 2 days after the surgery, then twice daily for 5 days (see below).

### 2.5 EEG recordings

Ten days after EEG electrode implantation surgery, animals were connected to the EEG recording system (Profusion 5, E‐series‐Amp, Compumedics, Australia) for a 24‐h baseline period. EEGs were unfiltered and digitized at 512 Hz. After each treatment, EEG recordings continued overnight for 24 h.^20^ The EEG cable was then detached, and the animal was returned to its home cage. This procedure was repeated every 7 days.

### 2.6 EEG analysis

EEGs were analyzed by two investigators who were blinded to treatment condition, using Assyst, an automated seizure detection software (Kaoskey, Australia).^19^ The events detected by Assyst were confirmed visually and independently by two experts using GAERS' standard criteria for the classification of seizures.^21^ The number of seizures, total seizure duration (cumulative time spent in seizures), and mean seizure duration were analyzed in 30‐min time intervals during the first 90‐min post‐treatment. The total number of seizures that occurred during the 90 min before huperzine A administration was analyzed in 30‐min intervals to determine potential pretreatment between‐group differences and the percentage change from baseline after treatment. To assess treatment effects over 24 h, these variables were also analyzed in 60‐min intervals at 6, 12, and 24 h post‐treatment and cumulatively over the 24‐h period before (baseline) and after each treatment administration.

### 2.7 Adverse effects scale

Animals were examined daily with a neurological adverse effects scale and a battery of tests that included scoring for sedation, ataxia, and muscle tone.^19^ Sedation was evaluated as (1) slightly reduced forward locomotion; (2) reduced locomotion with rest periods in between (partly with closed eyes); (3) reduced locomotion with more frequent rest periods (4) no forward locomotion, animal sits quietly (closed or open eyes); and (5) sleeping, animal can wake up (open eyes) when startled.^19^ Muscle tone was estimated by palpation of the abdomen.^19^ Measures of general health including weight, hydration, and grooming status also were monitored.^19^ These were completed daily by researchers blinded to the treatment groups. For each animal, behavior was evaluated within the home cage, and movement was observed on a level surface as well as on the wired cage lids.

### 2.8 Sample size and statistical analysis

Based on previous studies with GAERS,^18^, ^21^ to detect a ≥ 30% decrease in seizures for any treatment, with alpha set at 0.05 (two‐tailed) and beta set at 0.20 (80% power), an estimated 10 rats were required.

The Shapiro–Wilk test was used to evaluate the normal distribution of our data. Seizure data acquired during the 90‐min period (and evaluated in 30‐min blocks) immediately after treatment administration were analyzed with a two‐way repeated measures ANOVA (primary outcome). Tukey's post hoc tests were conducted to determine any pairwise differences between huperzine A and vehicle or positive control for the 30‐min intervals at 30, 60, and 90 min following treatment. One‐way analysis of variance (ANOVA) was used to analyze seizures across treatment groups during the baseline period (90 min immediately prior to injection). When the Shapiro–Wilk test demonstrated that the data were not normally distributed, outcomes were instead analyzed using Kruskal–Wallis one‐way ANOVA on ranks, followed post hoc by Dunn's multiple comparison test (e.g., analysis across treatments for the 1‐h intervals at 6, 12, and 24 h post‐treatment, and analysis of consecutive 30‐min post‐treatment intervals within treatment groups). The 90‐min pre‐ and post‐treatment interval and 24‐h pre‐ and post‐treatment interval were compared for each treatment using paired Student's *t*‐tests. Additionally, the relative reduction in seizures during the 90 min after treatment was calculated relative to the mean total number of seizures during the 90 min before treatment (i.e., 100% minus the percentage of seizures after treatment). Analyses are reported as mean and standard error of the mean (SEM) with statistical significance set at *p* < 0.05. GraphPad Prism 9 (GraphPad Software, Inc. USA) and SigmaPlot v14.0 (Systat Software, USA) were used for statistical analyses.

## 3 RESULTS

### 3.1 Huperzine A appeared well tolerated after a bolus dose

The three huperzine A doses (0.3, 1.0, and 3.0 mg/kg, i.p.) produced mild, transient, and dose‐dependent sedative effects. The positive control, ethosuximide (100 mg/kg, i.p.) produced similar effects (Table 1). No ataxia, changes in muscle tone, weight, or overall health were observed. No typical cholinergic side effects such as lacrimation, salivation, urination, or defecation were observed at the doses and route of administration used in the study.

**TABLE 1:** Summary of neurological adverse events.

Time of assessment (after dosing)								
Treatment dose (route)	Observations	5 min	10–15 min	30 min	1 h	2 h	3 h	6 h
Huperzine A 0.3 mg/kg (i.p.)	General^a^	No noticeable changes in behavior	Chattering behavior	Chattering behavior	Minimal chattering	Normal behaviors^b^	Normal behaviors^b^	Normal behaviors^b^
	Sedation^c^	Active and alert	++++	+++	++++	Active and alert	Active and alert	Active and alert
Huperzine A 1.0 mg/kg (i.p.)	General^a^	No noticeable changes in behavior	Porphyrin staining around eyes; chattering	Porphyrin staining around eyes; chattering	Porphyrin staining around eyes; chattering	Porphyrin staining around eyes; minimal chattering	Normal behaviors^b^	Normal behaviors^b^
	Sedation^c^	Active and alert	++++	++++	++++	+	Active and alert	Active and alert
Huperzine A 3.0 mg/kg (i.p.)	General^a^	No noticeable changes in behavior	Porphyrin staining around eyes	Porphyrin staining around eyes	Porphyrin staining around eyes	Porphyrin staining around eyes; minimal chattering	Normal behaviors^b^	Normal behaviors^b^
	Sedation^c^	Active and alert	++++	+++	+++	Active and alert	Active and alert	Active and alert
Ethosuximide 100 mg/kg (i.p.)	General^a^	Porphyrin staining around eyes	Porphyrin staining around eyes	Porphyrin staining around eyes	Porphyrin staining around eyes	Porphyrin staining around eyes	Porphyrin staining around eyes	Normal behaviors^b^
	Sedation^c^	Active and alert	++++	++++	++++	+++	++	Active and alert
Vehicle 0.9% NaCl pH 5.0 (i.p.)	General^a^	Porphyrin staining around eyes; normal behaviors^b^	Porphyrin staining around eyes; normal behaviors^b^	Porphyrin staining around eyes; normal behaviors^b^	Porphyrin staining around eyes; normal behaviors^b^	Normal behaviors^b^	Normal behaviors^b^	Normal behaviors^b^
	Sedation^c^	Active and alert	Active and alert	Active and alert	Active and alert	Active and alert	Active and alert	Active and alert

^a^ Measures of general health, including weight, hydration, and grooming status, were monitored.^19^

^b^ Normal behaviors include grooming, eating, and rearing.

^c^ Sedation was evaluated as (+) slightly reduced forward locomotion, (++) reduced locomotion with rest periods in between (partly with closed eyes), (+++) reduced locomotion with more frequent rest periods (partly with closed eyes), (++++) no forward locomotion; animal sits quietly with closed or open eyes, and (+++++) sleeping; animal can wake up (open eyes) when startled.^19^

### 3.2 Significant seizure reduction with huperzine A 3.0 mg/kg

A one‐way ANOVA showed no statistically significant baseline differences between groups in the total number of seizures during the 90‐min periods before each treatment (F_[5,54]_ = 1.71; *p* = 0.148). Figure 1 shows representative EEG recordings of the interictal and SWD recordings at baseline for vehicle‐treated animals (Figure 1B) and post‐treatment for huperzine A 3.0 mg/kg (Figure 1C) or ethosuximide (Figure 1D).

**FIGURE 2:**
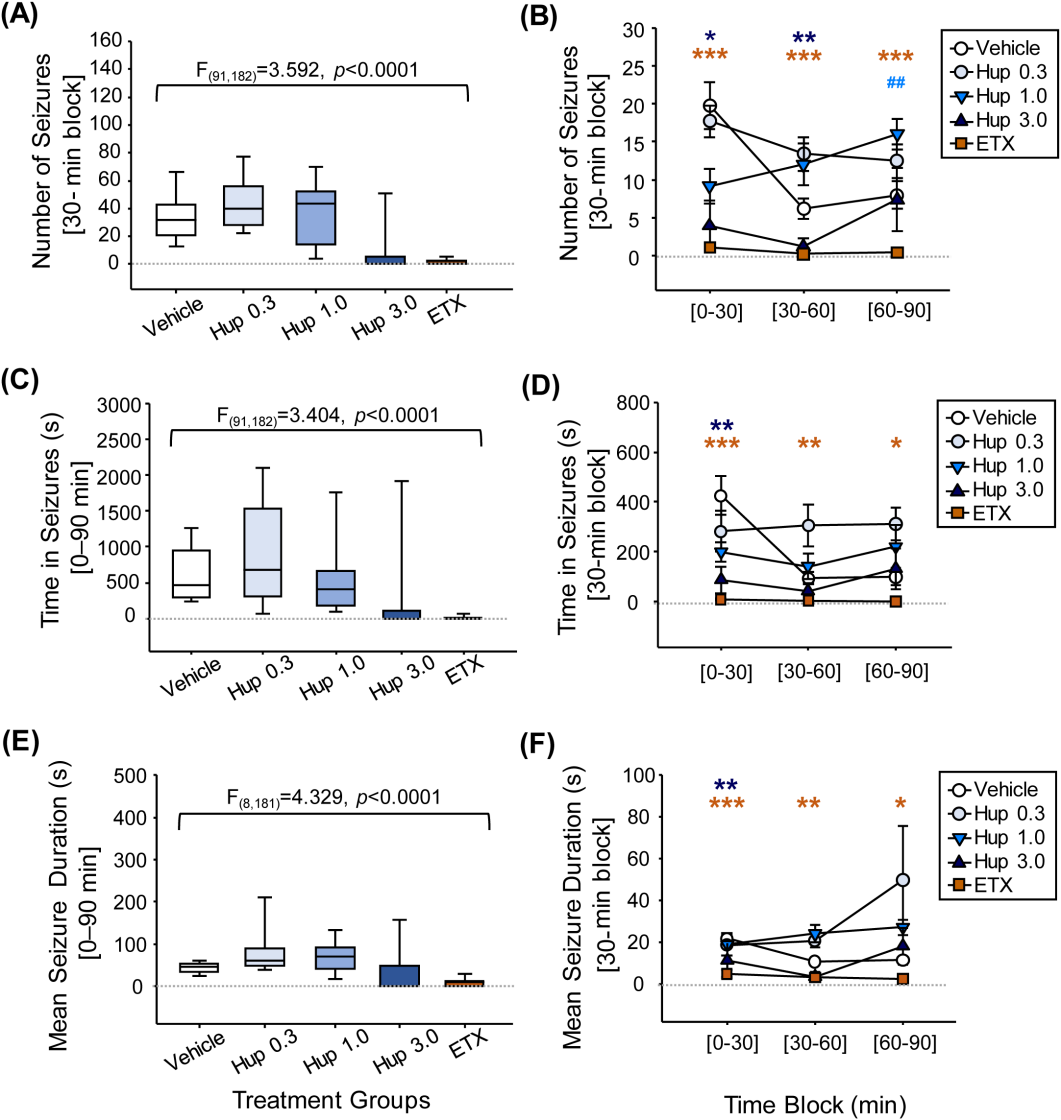
A single dose of huperzine A (3.0 mg/kg) significantly reduced seizures in GAERS over the initial 90 min after administration. (A, C, and E) Box plots summarize the data over the first 90 min after i.p. administration of vehicle; huperzine A (Hup) 0.3, 1.0, or 3.0 mg/kg; or ethosuximide (ETX) 100 mg/kg. F values correspond to effects of treatments by time in a two‐way ANOVA analysis. (B, D, and F) Time course over the first 90 min by 30‐min intervals after treatment. Data represent mean ± standard error of the mean (SEM). Significance was set at *p* < 0.05 in the two‐way repeated measures ANOVA (total 90‐min period) followed by Tukey's post hoc analysis (30‐min intervals), where *p < 0.05, **p < 0.01, and ***p < 0.001, represent significant differences favoring treatment, and ^##^ *p* < 0.01 represents a significant difference favoring vehicle for the indicated treatment for that interval.

Two‐way ANOVA showed statistically significant differences among treatment groups on all three outcomes: number of seizure events (*F*_(91,182)_ = 3.592; *p* < 0.0001; Figure 2A), total time spent in seizures (*F*_(91,182)_ = 3.404; *p* < 0.0001; Figure 2C), and mean seizure event duration (*F*_[8,181]_ = 4.329; *p* < 0.0001; Figure 2E) during the first 90‐min period after drug administration.

Because there were no significant differences between the two vehicle administration periods, only the first vehicle period was used for post hoc comparisons. Tukey's post hoc tests (Figure 2B,D,F) showed statistically significant effects for huperzine A 3.0 mg/kg relative to vehicle at 30 min for all three outcomes and at 60 min for reduction in seizure number. Statistically significant effects for ethosuximide relative to vehicle were observed at 30, 60, and 90 min for all three outcomes. Differences between huperzine A 3.0 mg/kg and ethosuximide were not statistically significant for any comparison. Lower doses of huperzine A (0.3, 1.0 mg/kg) did not differ significantly versus vehicle for any seizure measure, except that a relative increase in seizure number (*p* = 0.0031) was observed for huperzine A 1.0 mg/kg during the third 30‐min interval (60–90 min) after treatment (Figure 2B).

Likewise, post hoc paired *t*‐tests comparing the 90 min before and after each treatment showed significant post‐treatment reductions in mean seizure counts relative to baseline for the huperzine A 3.0 mg/kg dose (*p* < 0.001) and for ethosuximide (*p* < 0.001) but not for the vehicle (*p* = 0.611), and lower huperzine A doses (*p* = 0.792 for 0.3 mg/kg and *p* = 0.539 for 1.0 mg/kg). Overall, huperzine A 3.0 mg/kg reduced seizure counts by 87.3% ± 5.8% and ethosuximide reduced seizure counts by 95.7% ± 1.1% during the first 90 min after treatment relative to pretreatment baseline.

Based on the finding of higher seizure count relative to vehicle for the 1.0 mg/kg huperzine A dose at the 60‐ to 90‐min post‐treatment interval, we further evaluated homogeneity of seizure counts for vehicle and for the 1.0 mg/kg dose across the three 30‐min post‐treatment intervals. Despite similar mean ± SEM seizure counts during the overall 90‐min periods before and after each treatment (37 ± 4 and 34 ± 4 for vehicle and 43 ± 6 and 37 ± 6 for the 1.0 mg/kg huperzine A dose), one‐way ANOVA on ranks showed significant differences across the 30‐min post‐treatment intervals for vehicle (*H* = 15.8335, *p* < 0.001) with post hoc comparison showing more seizures during the initial 0–30 min interval relative to the 30–60 min (*p* < 0.001) and 60–90 min (*p* = 0.006) intervals. In contrast, one‐way ANOVA on ranks was not significantly different across these three intervals for the 1.0 mg/kg huperzine A dose (*H* = 5.102, *p* = 0.078).

### 3.3 Seizure suppression over the 24‐h period post‐administration

**FIGURE 3:**
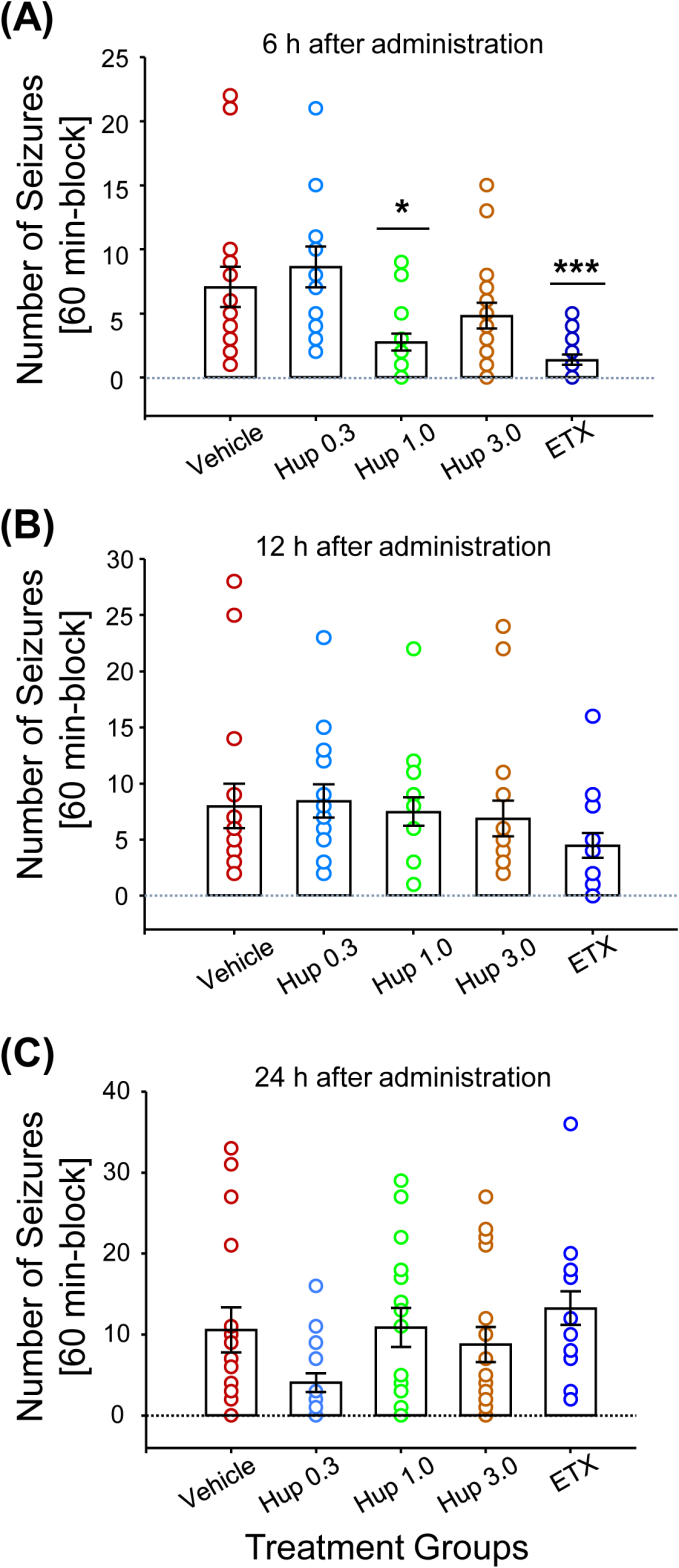
Effect of single administration of huperzine A (Hup) and ethosuximide (ETX) on number of seizures during the 60‐min interval starting at (A) 6 h, (B) 12 h, and (C) 24 h following treatment. Each circle represents the number of seizures observed in a single animal during the specified 60‐min interval following treatment. All animals receiving each treatment are represented on the graph. Bars represent the mean ± standard error of the mean (SEM). Data were analyzed by Kruskal–Wallis one‐way ANOVA on ranks followed by Dunn's multiple comparison test between active and vehicle‐treated groups at each time point (*p = 0.05 and ***p < 0.001 based on Dunn's test). ETX, ethosuximide 100 mg/kg; Hup 0.3, huperzine A 0.3 mg/kg; Hup 1.0, huperzine A 1.0 mg/kg; Hup 3.0, huperzine A 3.0 mg/kg.

To evaluate the time course of huperzine A effects on the number of seizures up to 24 h after dosing, snapshots of 1‐h intervals starting at 6, 12, and 24 h after injection were analyzed (Figure 3). The one‐way ANOVA on ranks showed significant treatment differences at 6 (*H* = 27.154; *p* < 0.001) and 24 h (*H* = 12.886; *p* = 0.012). Dunn's post hoc analysis showed that apart from a significant seizure reduction for huperzine A 1.0 mg/kg (*p* = 0.05) and ethosuximide (*p* < 0.001) at 6 h versus vehicle (Figure 3A), there were no statistically significant differences for any huperzine A dose versus ethosuximide or vehicle, nor for ethosuximide versus vehicle at any other time point (Figure 3B,C).

**FIGURE 4:**
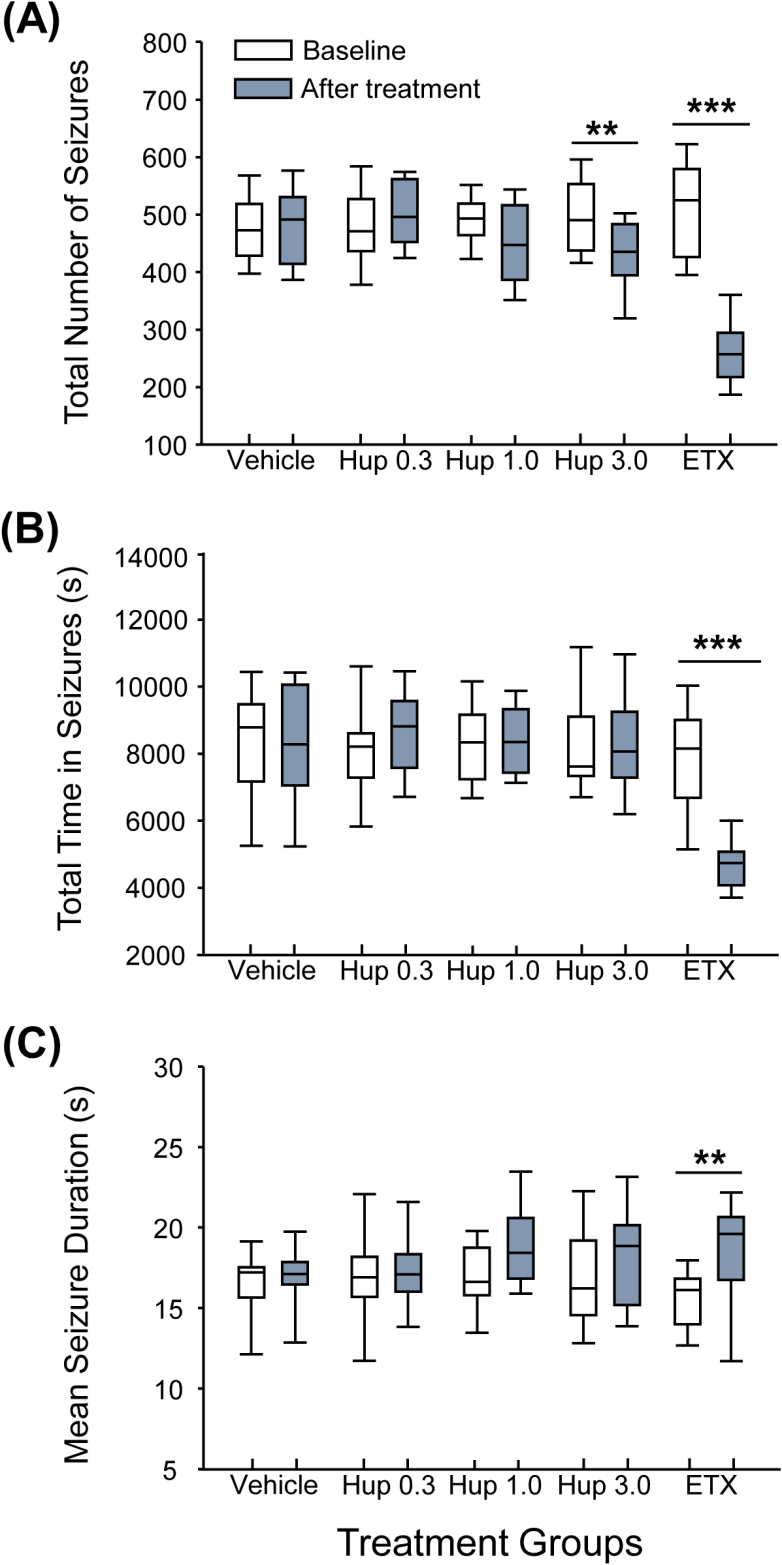
Effect of treatments in the GAERS model 24 h before and after administration. (A) Box plots of total number of seizures. (B) Total time spent in seizures. (C) Mean seizure duration during the 24‐h period before (baseline) and after each treatment administration. Pre‐ and post‐treatment intervals were compared using two‐tailed Student's *t*‐tests (**p < 0.01, ***p < 0.001). ETX, ethosuximide 100 mg/kg; GAERS, Genetic Absence Epilepsy Rat from Strasbourg; Hup 0.3, huperzine A 0.3 mg/kg; Hup 1.0, huperzine A 1.0 mg/kg; Hup 3.0, huperzine A 3.0 mg/kg.

EEG analysis of seizure activity during the cumulative 24‐h periods before (baseline) and after treatment showed that huperzine A 3.0 mg/kg significantly reduced (*p* = 0.004) the total number of seizures relative to baseline (Figure 4A) with no significant changes in the overall time spent in seizures (Figure 4B) or mean seizure duration (Figure 4C). Ethosuximide 100 mg/kg significantly decreased the total number and overall time spent in seizures (*p* < 0.001) but significantly increased mean duration of seizure events (*p* < 0.01) relative to baseline (Figure 4C).

## 4 DISCUSSION

This blinded, randomized, vehicle‐ and positive‐controlled study demonstrated that a single dose of huperzine A 3.0 mg/kg was well tolerated and significantly suppressed seizures in the GAERS model of GGE with absence seizures. At the 3.0 mg/kg dose, huperzine A appeared well tolerated, with only minimal and transient post‐dose sedation. The seizure suppressant effect was not significantly different (and appeared visually similar) to ethosuximide during the first 60 min after drug administration. Consistent with huperzine A time of maximal concentration in the rat brain after i.p. administration (T_max_ = 60 min), and its relatively short plasma half‐life (T_1/2_ = 2 h), the seizure suppressant effect of this dose versus vehicle was not sustained at later time intervals, although (unlike the lower doses of huperzine A) it was still not statistically different from ethosuximide (T_1/2_ = 12 h). This suggests some lingering seizure suppression with waning drug concentrations, consistent with the reduction in total number of seizures during the 24 h period post‐treatment.

To this end, it is difficult to interpret the increase in seizure frequency at the 60–90‐min post‐treatment interval, and the paradoxical decrease in seizure frequency at the 6‐h post‐treatment interval for the huperzine A 1.0 mg/kg dose relative to vehicle. Because the overall number of seizures did not change significantly from baseline during the 90‐min post‐treatment interval, we can only postulate that these anomalies may be due to chance. Natural irregularities of seizure occurrence in the GAERS model would be more likely to yield non‐uniform counts during short time frames. Notably, within the vehicle group, seizure rates were significantly lower during the 30–60‐min and 60–90‐min post‐treatment intervals relative to the 0–30‐min interval (see below).

Potentially confounding the ability to show seizure suppression within the first 90 min post‐dosing was the observable reduction in seizures in the vehicle group. The effect observed in the first 30 min after treatment was presumably related to the impact of animal handling and injection on seizure frequency. SWDs in GAERS commonly occur in quiet wakefulness periods and can be triggered after a stressful stimulus.^21^ Likewise, SWDs in people with absence epilepsy can be triggered after stress, hyperventilation, and sleep deprivation and also can occur within reduced vigilance states, corresponding to quiet wakefulness or somnolence in GAERS.^22^

The mechanism of huperzine A seizure suppression is not entirely clear. Huperzine A increases extracellular concentrations of ACh by inhibiting AChE. In turn, this is thought to increase cholinergic‐mediated augmentation of inhibitory pathways; however, a direct effect on GABA neurotransmission has not been proven.^3^, ^5^ Moreover, increases in GABA have been shown to promote SWDs in GAERS,^2^ suggesting that the reduction in number of seizures by huperzine A might be mediated by a different mechanism. Huperzine A also may enhance cholinergically governed anti‐inflammatory pathways,^23^ including NLRP3/caspase‐1 inhibition,^24^ and tumor necrosis factor and interleukin‐1β (IL‐1β) reduction.^25^ Blocking caspase‐1 may prevent seizures, while IL‐1β expression in the somatosensory cortex is ictiogenic in GAERS.^26^ Interestingly, huperzine A provides potent and specific inhibition of AChE in the somatosensory cortex, the brain region where seizures originate in GAERS.^27^, ^28^ Still, anti‐inflammatory mechanisms may not explain the rapid onset of effect in the GAERS model. Additional research is needed to evaluate chronic administration of huperzine A on seizure suppression in the GAERS model and in modulating the pathways mentioned above.

### 4.1 Strengths/limitations

This is the first study to evaluate the effects of an AChE inhibitor in the GAERS model. A strength of the study was its randomized, blinded, crossover design, so each animal served as its own control. In addition, multiple doses of huperzine A were tested, and the effects of huperzine A were compared with negative (vehicle) and positive (ethosuximide) control groups. A study limitation was the short (single‐dose) duration of treatment; thus, the effects of chronic treatment, as would be used in patients, could not be evaluated. Additionally, although the effects of huperzine A were measured relative to the known effect of ethosuximide as a positive control, this was not a non‐inferiority design, meaning the study design was not sufficient to demonstrate whether huperzine A is at least as effective as ethosuximide. Therefore, equality to ethosuximide cannot be concluded despite the lack of statistically significant differences for seizure outcomes between huperzine A and ethosuximide.

### 4.2 Clinical relevance and future directions

While the mechanisms by which huperzine A exerts its anti‐seizure effects are beyond the scope of this study, we hypothesize that this can be due to three potential pathways. First is the enhancement of cholinergic anti‐inflammatory pathways,^23^ including NLRP3/caspase‐1 inhibition,^24^ and tumor necrosis factor and IL‐1β reduction.^25^ Evidence shows that huperzine A‐dependent AChE inhibition is specific and more potent in the somatosensory cortex,^27^ where seizures are generated in the GAERS model.^28^ Moreover, blocking caspase‐1 prevents seizures while IL‐1β expression in the somatosensory cortex is ictiogenic in GAERS.^26^ Second, oxidative stress is widely recognized as a contributor to the development of seizures and epilepsy, and huperzine A administration has been shown to reduce oxidative stress and increase glutathione.^25^ In fact, a recent multiomics study in GAERS showed that glutathione expression was decreased in the somatosensory cortex in GAERS.^29^ Third, cholinergic activity mediated by huperzine A could potentially restore excitatory/inhibitory balance by stimulating cholinergic receptors in different neuronal subtypes and glial cells in relevant brain regions. Stimulation of cholinergic receptors expressed in inhibitory interneurons^30^ could potentiate inhibitory mechanisms or reduce neurotransmitter release in glutamatergic axon terminals.^31^ Stimulation of nicotinic receptors can increase glutamate uptake to reduce extracellular glutamate.^32^ Importantly, increases in GABA promote SWDs in GAERS.^33^ Similarly, it is typically observed that many GABAergic anti‐seizure drugs exacerbate seizures in patients with GGE.^11^ The robust suppression of a single‐dose huperzine A in this model provides evidence that huperzine A acts differently than typical GABAergic drugs and could provide clinical benefit to patients with GGE.

This study demonstrated huperzine A robustly suppressed absence‐like seizures in the GAERS model of GGE, with efficacy similar to ethosuximide in the early period after administration. Coupled with the efficacy demonstrated in other murine seizure models,^9^, ^34^ the effects in GAERS suggest huperzine A may have broad‐spectrum anti‐seizure activity, with potential to treat absence seizures in GGE, as well as acquired focal epilepsies.

## CONFLICT OF INTEREST STATEMENT

This study was supported by a Research Grant from Supernus Pharmaceuticals, Inc. Pablo Casillas‐Espinosa and Terence J. O'Brien received a research grant from Supernus Pharmaceuticals, Inc. to perform the experiments described in this manuscript. Rui Li and Crystal Li have no conflicts of interest to declare. Jennie Garcia‐Olivares, Chungping Yu, and Andrea E. Formella are employees of Supernus Pharmaceuticals, Inc.

## ETHICS STATEMENT

We confirm that we have read the Journal's position on issues involved in ethical publication and affirm this report is consistent with those guidelines.

## Data Availability

Access to datasets generated or analyzed for this publication can be made available to independent researchers after receipt of a valid research proposal, data analysis plan, and summary of researcher qualifications. Requests may be submitted to Supernus Pharmaceuticals, Inc. at medicalaffairs@supernus.com. Provision of data is contingent on business feasibility and execution of a data use agreement.
